# Human immunodeficiency virus integrase inhibitors efficiently suppress feline immunodeficiency virus replication *in vitro *and provide a rationale to redesign antiretroviral treatment for feline AIDS

**DOI:** 10.1186/1742-4690-4-79

**Published:** 2007-10-30

**Authors:** Andrea Savarino, Mauro Pistello, Daniela D'Ostilio, Elisa Zabogli, Fabiana Taglia, Fabiola Mancini, Stefania Ferro, Donatella Matteucci, Laura De Luca, Maria Letizia Barreca, Alessandra Ciervo, Alba Chimirri, Massimo Ciccozzi, Mauro Bendinelli

**Affiliations:** 1 Dept. of Infectious, Parasitic and Immune-mediated Diseases, Istituto Superiore di Sanità, Viale Regina Elena, 299, 00161, Rome, Italy; 2 Dept. of Experimental Pathology, Univ. of Pisa, Via San Zeno 37, 56127 Pisa, Italy; 3 Pharmaco-chemical Dept., Univ. of Messina, Viale Annunziata, 98168 Messina, Italy

## Abstract

**Background:**

Treatment of feline immunodeficiency virus (FIV) infection has been hampered by the absence of a specific combination antiretroviral treatment (ART). Integrase strand transfer inhibitors (INSTIs) are emerging as a promising new drug class for HIV-1 treatment, and we evaluated the possibility of inhibiting FIV replication using INSTIs.

**Methods:**

Phylogenetic analysis of lentiviral integrase (IN) sequences was carried out using the PAUP* software. A theoretical three-dimensional structure of the FIV IN catalytic core domain (CCD) was obtained by homology modeling based on a crystal structure of HIV-1 IN CCD. The interaction of the transferred strand of viral DNA with the catalytic cavity of FIV IN was deduced from a crystal structure of a structurally similar transposase complexed with transposable DNA. Molecular docking simulations were conducted using a genetic algorithm (GOLD). Antiviral activity was tested in feline lymphoblastoid MBM cells acutely infected with the FIV Petaluma strain. Circular and total proviral DNA was quantified by real-time PCR.

**Results:**

The calculated INSTI-binding sites were found to be nearly identical in FIV and HIV-1 IN CCDs. The close similarity of primate and feline lentivirus IN CCDs was also supported by phylogenetic analysis. In line with these bioinformatic analyses, FIV replication was efficiently inhibited in acutely infected cell cultures by three investigational INSTIs, designed for HIV-1 and belonging to different classes. Of note, the naphthyridine carboxamide INSTI, L-870,810 displayed an EC_50 _in the low nanomolar range. Inhibition of FIV integration *in situ *was shown by real-time PCR experiments that revealed accumulation of circular forms of FIV DNA within cells treated with L-870,810.

**Conclusion:**

We report a drug class (other than nucleosidic reverse transcriptase inhibitors) that is capable of inhibiting FIV replication *in vitro*. The present study helped establish L-870,810, a compound successfully tested in human clinical trials, as one of the most potent anti-FIV agents ever tested *in vitro*. This finding may provide new avenues for treating FIV infection and contribute to the development of a small animal model mimicking the effects of ART in humans.

## Background

Animal models have been essential for preclinical testing of antiretroviral strategies. Macaques infected with the simian/human immunodeficiency virus (SHIV) chimera are a well established model, which recently provided the first proof of concept for an antiretroviral effect of integrase strand transfer inhibitors (INSTIs) *in vivo *[1]. The simian model can be used, however, only by institutions able to support the high costs of primate facilities. Moreover, SHIV-infected macaques may represent an ethical problem, and the obstacles to obtaining permission to conduct research in primates have recently been intensified [2].

Feline immunodeficiency virus (FIV)-infected cats have been proposed as an alternative/complementary animal model for HIV-1/AIDS [3,4]. Cats are easier to house and maintain, due to long adaptation to coexistence with humans [5]. Moreover, easy access to naturally infected animals could allow a better estimate of the impact of a treatment on different circulating viral strains.

FIV is phylogenetically (though not antigenically) related to HIV-1 [3]. Although vaccines designed for FIV cannot directly be transferred to HIV-1, the feline model may find an application in preliminarily testing the general validity of an approach to vaccination [6], or to test the feasibility of lentiviral eradication strategies.

A major limitation of the feline model is, however, the absence of treatments mimicking the sustained effects of combined antiretroviral therapies (ART) in humans. Similarly to HIV-1, FIV was shown to respond to nucleosidic reverse transcriptase (RT) inhibitors (NRTIs) [7,8]. However, FIV is not inhibited by non-nucleosidic RT inhibitors (NNRTIs) [8,9] and protease inhibitors (PIs) acting on HIV-1 [8,10], although the latter drug class was found to inhibit a wide range of non-HIV-1 targets [11-14]. The absence of at least two drug classes inhibiting FIV hampered the possibility of using combination ART in the feline model.

INSTIs represent a highly promising new drug class for HIV-1/AIDS, and at least three such drugs have shown potent antiretroviral effects in human clinical trials [1,15,16]. The anti-HIV-1 potency of INSTIs at least equals that of NNRTIs and PIs [1,15]. FIV IN was characterized in the last decade [17,18]. Similar to HIV-1 IN, the FIV protein catalyzes 3' end processing, 3'end joining and disintegration of proviral DNA [17,18] (the biological significance of the last of these reactions is as yet unknown [1]). The reactions are absolutely dependent on divalent cations, Mn^++ ^or Mg^++ ^[17]. The substrate specificity of FIV IN is relaxed, and the protein was found to be active on oligonucleotides containing sequences derived from the U5 end of HIV-1 and murine leukemia virus (MLV) [17]. The enzyme structure of FIV IN is similar to that of HIV-1 IN; and it is organized in C- and N- terminal domains, and a catalytic core domain (CCD). The C-terminal domain is likely to be involved in target (*i.e*., cellular) DNA binding. In contrast to what was reported for other retroviral INs, deletion of the C-terminal domain does not abrogate the catalytic activities of FIV IN, although the efficiency of the 3' processing and strand transfer reactions is decreased in the truncated forms. Similar to other retroviral INs, FIV IN is likely to act as a multimer [17]. At this time, the three-dimensional (3D) structure of FIV IN is unknown, as is the response of FIV to INSTIs. In the present paper, we focus our attention on the CCD, because it is the protein portion principally involved in binding of INSTI drugs to proviral DNA/IN complexes, as shown in previous studies on HIV-1 IN [1,19-22].

We here describe the first three-dimensional (3D) model for FIV IN CCD, and show that the catalytic site of FIV IN is nearly identical to that of the HIV-1 ortholog. Amino acids calculated to be involved in drug binding are highly conserved between HIV-1 and FIV INs. Moreover, INSTIs inhibit FIV replication in cell cultures as efficiently as HIV-1 replication. The possibility of targeting a second FIV enzyme with antiretroviral drugs may provide a basis for the design of an ART for FIV.

## Results and discussion

### Clustering of lentiviral enzymes

**Figure 1:**
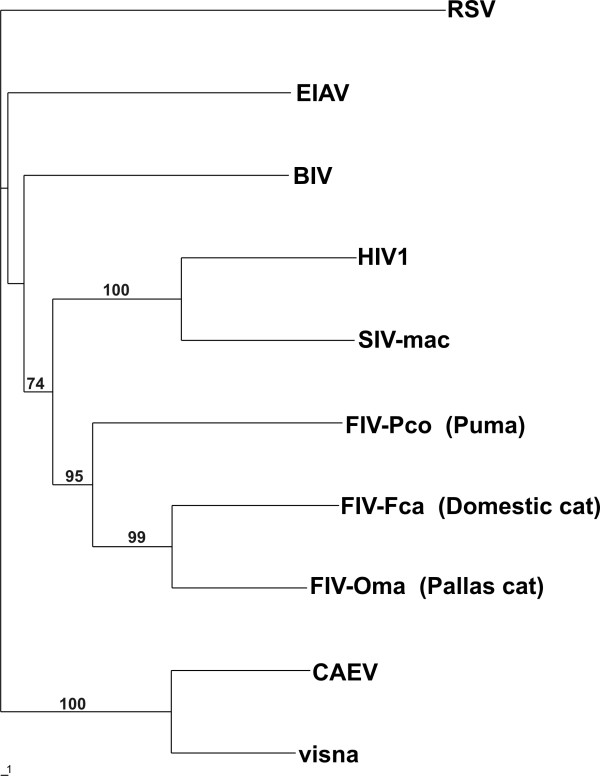
**Phylogenetic analysis of lentiviral integrase core domains**. Bootstrap values > 70% are shown. Rous sarcoma virus (RSV) [PDB: 1ASV] served as outgroup. Sequence adopted: equine infectious anemia virus (EIAV) [Swiss-Prot: P11204]; Jembrana disease virus, belonging to the bovine immunodeficiency virus (BIV) group [REFSEQ: NC_001654.1]; human immunodeficiency virus type-1 (HIV-1) [PDB: 1BL3C]; simian immunodeficiency virus, host: macaque (SIV-mac) [PDB: 1C6VC]; feline immunodeficiency virus, host: domestic cat (FIV-Fca) [REFSEQ: NP_040973.1]; feline immunodeficiency virus, host: Pallas' cat (FIV-Oma) [GenBank: AAB49923]; puma lentivirus (FIV-Pco) [GenBank: AAA67168]; caprine arthritis-encephalitis virus (CAEV) [Swiss-Prot: P33459]; visna lentivirus [Swiss-Prot: P23427].

To determine which of the non-primate lentivirus IN CCDs might have the closest similarity to the HIV-1 IN CCD, a phylogenetic analysis of the amino acid sequences of lentiviral IN CCDs was carried out. We chose to use amino acid rather than nucleic acid sequences because open-access databases do not report the IN CCD nucleic acid sequences for some important members of the *Lentivirus *genus. Moreover, our phylogenetic analysis was intended to analyze the similarities of the CCDs of the mature lentiviral proteins, rather than to reconstruct a phylogeny of the *Lentivirus *genus. We found that the IN CCDs of feline lentiviruses are more closely related to those of the HIV/SIV group than any other non-primate lentiviral IN CCDs (Fig. 1). This result is supported by the significant bootstrap values obtained (Fig. 1).

Previous analyses based on the entire *pol *gene or the entire *IN *region produced different results, showing the feline lentiviruses, ungulate lentiviruses and the HIV/SIV group as equally distant from one another [23,24]. The results of the present study are likely to be attributed the fact that 1) we used the isolated CCD; 2) amino acid sequences facilitate the discovery of similarities in the mature proteins by excluding silent mutations that may have occurred during phylogenesis. Be that as it may, the finding of a significant clustering of primate and feline lentivirus IN CCDs encouraged us to further analyze the similarities of HIV-1 and FIV IN CCDs.

### Amino acid conservation between HIV-1 and FIV integrases

Drug resistance studies and site-directed mutagenesis showed that mutation of any of five HIV-1 IN amino acids (*i.e*., T66, E92, F121, Q148, and N155) confers significant cross-resistance to INSTIs [1,25-27]. Drug resistance mutations N155H and Q148R were shown to hamper INSTI binding to HIV-1 IN, by either decreasing the affinity of IN/proviral DNA complexes for INSTIs (N155H) or affecting assembly of proviral DNA (Q148R) [27]. Previous computational simulations conducted by one of us suggest that T66, E92, F121, and N155 are involved in important interactions of HIV-1 IN with the antiretroviral drugs [22].

**Figure 2:**
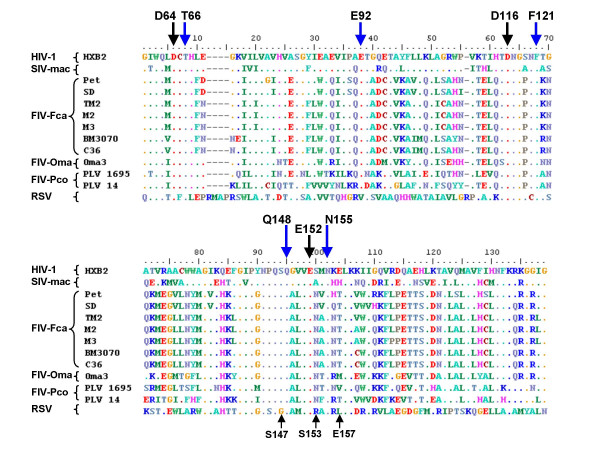
**Amino acid sequence alignment of the lentiviral integrase catalytic core domain (IN CCD)**. Amino acid sequences were aligned with BioEdit and alignments manually edited to eliminate gaps. FIV-Fca, FIV-Oma, and FIV-Pco refer to feline immunodeficiency viruses from domestic cat, Pallas' cat, and puma, respectively. The FIV-Fca clade is indicated by capital letters. The catalytic triad is marked by the black arrows. Blue arrows show the amino acids reported to confer significant cross-resistance to the major classes of IN strand transfer inhibitors. Small arrows show minor drug resistance mutations. Amino acid numbering refers to HIV-1 IN. The Pol IN CCD sequences aligned were from: immunodeficiency virus type-1 (HIV-1) [PDB: 1BL3C]; simian immunodeficiency virus, host: macaque (SIV-mac) [PDB: 1C6VC]; FIV-Fca: Petaluma (Pet) [REFSEQ: NP_040973.1], San Diego (SD) [Swiss-Prot: :P19028], TM2 [GenBank: AAA43071], BM3070 [GenBank: AAM13444], C36 [GenBank: AAT12494]; FIV-Oma: Oma-3 [GenBank: AAU20798.1]; FIV-Pco: PLV-1695 [GenBank: ABB29307.1] and PLV-14 [GenBank: AAA67168.1]. M2 and M3 are local field isolates of FIV-Fca, clade B (Pistello et al., 1997, sequences being submitted to GenBank).

To analyze differences between HIV-1 and feline lentiviruses at these amino acid positions, we performed alignments of the HIV-1 IN CCD sequence with selected sequences of INs from highly divergent feline lentiviruses. The amino acid positions corresponding to T66, E92, F121, Q148, and N155 in HIV-1 IN were found to be highly conserved between HIV-1 and feline lentiviruses (Fig. 2). These amino acids are also conserved in simian immunodeficiency virus (SIV) IN (susceptible to INSTIs [26]) but not in Rous sarcoma virus (RSV) IN (which is not inhibited by INSTIs [26]). As regards the less important primary drug resistance mutations of HIV-1 IN, *i.e*. S147, S153 and E157, only the amino acid corresponding to HIV-1 IN S147 is conserved in FIV IN. These amino acids, however, do not confer cross resistance to the different INSTIs and were shown to confer low-level resistance only to the quinolonic INSTI, namely elvitegravir [25]. Moreover, apart from S147, these amino acids are not even conserved in SIVmac IN, which is known to be fully susceptible to important classes of INSTIs such as diketo acids and naphthyridine carboxamides [26].

Recent phylogenetic analyses suggest that feline lentiviruses are monophyletic [28]. Therefore, the amino acid conservation shown by the highly divergent sequences examined in the present study most likely includes the majority of feline lentiviruses. For example, the key residues for response to INSTIs are conserved not only in the different domestic cat (*Felis sylvestris catus*) sequences analyzed, but also in sequences from Pallas' cat (*Otocolobus manul*) and mountain lion (*Puma concolor*) (Fig. 2). These sequences belong to feline lentiviruses from lineages that are distinct from viruses circulating in domestic cats [28].

We conclude that FIV and HIV-1 INs share conservation of some amino acid residues important for response to INSTIs. This finding *per se*, however, could not be used as evidence for susceptibility of FIV to INSTIs. Indeed, other amino acids that are not conserved between HIV-1 and FIV may contribute to conformational differences and be capable of limiting susceptibility to INSTIs.

### *In-silico *modeling of FIV integrase catalytic core domain complexed with the transferred strand of proviral DNA and molecular docking of antiretroviral drugs

Starting with conservation of important HIV-1 and FIV IN residues, we built a 3D model of IN CCD of the Petaluma strain of FIV (FIV-Pet) by homology with HIV-1 IN CCD. Homology modeling of FIV IN CCD based on a crystal structure of its HIV-1 counterpart was encouraged by the high level of conservation of the 3D structures of the catalytic sites of retroviral INs and the related enzyme Tn5 transposase. Homology modeling is a viable technique in the absence of crystal structures of a given protein, and helps in predicting the 3D structure of a macromolecule with unknown structure (target) by comparing it with a known template from another, structurally highly similar, macromolecule. In general, 30% sequence homology is required for generating useful models. Here, the sequence identity between target and template was 44%. As a template structure, we chose the subunit C of the structure of HIV-1 IN CCD described by Maignan *et al*. [29] Similarly to all HIV-1 IN structures complexed with metals, the structure of Maignan *et al*. presents only one of the (likely) two metal ions in the catalytic cavity, but, differently from other published HIV-1 IN CCD structures, displays a well ordered catalytic triad [29]. Another reason for considering the structure of Maignan *et al*. for our homology modeling purpose was the presence of the entire flexible loop (amino acids 140–152) in chain C. The flexible loop is often absent from published IN CCD structures or in positions which likely do not reflect that assumed *in vivo*. In chain C of the structure of Maignan *et al*., the flexible loop connects two CCD subunits in a dimer that may have biological significance, as the distance between the two active sites corresponds to 18 Å, approximately one half turn of a Watson-Crick-Franklin DNA helix (*i.e*., the distance at which the two antiparallel strands of acceptor DNA are simultaneously nicked during strand transfer) [22]. Thus, the flexible loop is, in this case, likely to be in a position reflecting that assumed in pre-integration complexes [22].

**Figure 3:**
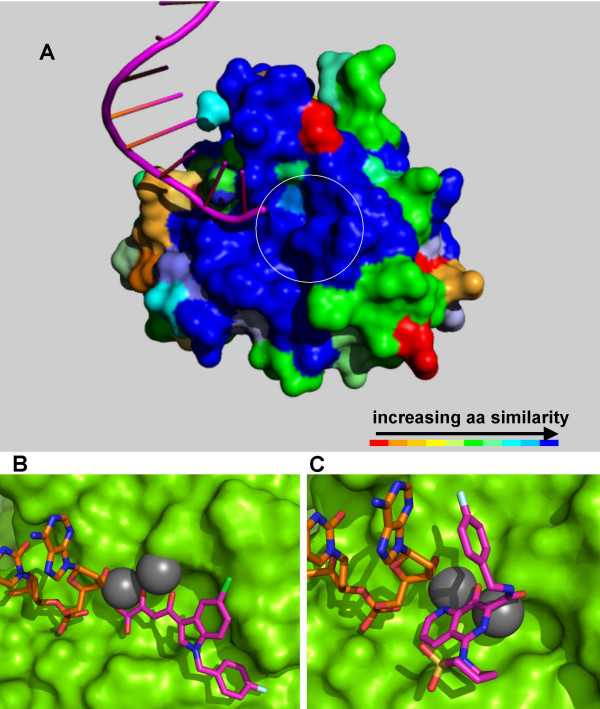
**Proposed binding mode of integrase strand transfer inhibitors (INSTIs) to FIV integrase**. Panel A: A three-dimensional model of FIV-Pet IN catalytic core domain in complex with the transferred strand of viral DNA c. The enzyme is colored by sequence similarity with its HIV-1 orthologue [PDB:1BL3]. The level of similarity was calculated by the Swiss PDB Viewer (SPDBV) software. The color scale is that adopted by SPDBV. The transferred strand of proviral DNA is shown in magenta. Similarity is maximal at the level of the INSTI binding site. The INSTI binding site (indicated by a circle) is that calculated by some of us in previous works [16,20]. Panels B-C: Docking of CHI1019 (panel B) and L-870,810 (panel C) at the catalytic cavity of FIV IN. The protein is shown as Connolly surface (in green). Ligands are shown in CPK (carbon backbone in magenta). The terminal dinucleotide of 3' processed proviral DNA is shown in CPK (carbon backbone in orange). Metals are shown as spheres (in gray). Images prepared using Pymol (see Ref. [50]).

The FIV-Pet IN CCD was thus modeled using chain C of the structure of Maignan *et al*. as a template. The resulting model was subjected to energy minimization, and Ramachandran analysis was done to validate the model. Results showed that the sequence of FIV-Pet IN CCD was consistent with the 3D folding of HIV-1 IN CCD: 95% of the residues were in Ramachandran-favored position and 5% were in Ramachandran-allowed positions [see Additional file 1]. When HIV-1 and FIV IN CCD structures were superimposed, all amino acids facing the catalytic cavity were similar, except for HIV-1 IN Y143, which is substituted with a glycine in FIV (Figs 2 and 3A).

As INSTIs were shown to require proviral DNA to bind to HIV-1 IN [1,27], a model for the FIV IN CCD complexed with the transferred strand of proviral DNA was prepared to simulate INSTI binding to the catalytic cavity of FIV IN. Briefly, the homology-based model for FIV IN CCD was superimposed to a crystal structure of Tn5 transposase complexed with transposable DNA [PDB: 1MM8] (the structural similarities between the catalytic cavities of Tn5 transposase and retroviral INs have been previously described [20,22,30]). The 3' filament of transposable DNA (corresponding to the transferred strand of retroviral DNA) and the metal ion coordinating the 3' DNA hydroxyl were transferred to the FIV IN CCD model. The terminal dinucleotide was manually corrected to 5'-CA-3' (*i.e*. the highly conserved dinucleotide at the 3' end of integrated lentiviral DNA; see Fig. 3A), and the DNA-coordinating Mn^++ ^ion was corrected to a Mg^++ ^type, *i.e*. the metal likely to be present *in vivo *[1]. The E152 sidechain was brought to metal-coordinating position, as previously described for a two-metal model of HIV-1 IN CCD [22]. The position of the second Mg^++ ^ion likely to be important for INSTI binding (*i.e*., that between residues corresponding to D64 and D116 of HIV-1 IN [1,20,22]) was deduced from the HIV-1 IN CCD structure of Maignan *et al*. [PDB: 1BL3].

**Figure 4:**
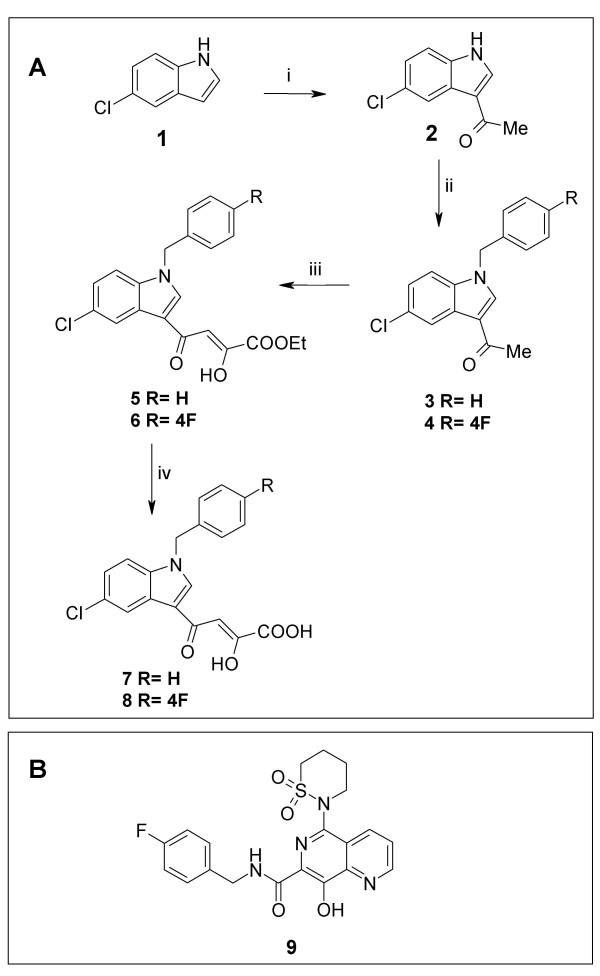
**Integrase strand transfer inhibitors adopted in the present study**. Panel A: Synthesis of CHI1010 (**7**) and CHI1019 (**8**). Reagents and conditions: i) AcCl, Et_2_AlCl, CH_2_Cl_2_, 0°C, 2 h. ii) benzyl or 4-fluorobenzyl bromide, NaH, DMF, 0°C, 30 min; iii) diethyl oxalate, dry C_2_H_5_ONa, THF, two separated steps in the same conditions: 50°C, 2 min, 250 W, 300 psi; iv) 2N NaOH, MeOH, rt, 1.5 h. Panel B: structure of Merck's compound L-870,810 (**9**).

Docking simulations of compounds (**8,9**), namely, respectively, CHI1019 and L-870,810 (see Fig. 4), were conducted using the genetic algorithm GOLD. These compounds are representative of two important classes of INSTIs. CHI1019 is a novel diketo acid, which was recently designed by some of us and shown to inhibit HIV-1 replication *in vitro *[31]. L-870,810 is a naphthyridine carboxamide developed by Merck researchers, which was the first INSTI to furnish proof of concept for an antiretroviral effect in humans [1,26]. We found that the structures of the investigational INSTIs allowed docking at the FIV IN catalytic cavity (Fig. 2B–C ). The INSTIs displayed high GOLD fitness scores (> 60; data not shown), which are in our experience significantly associated with enzyme inhibitory interactions [22]. We conclude that the calculated structure of the catalytic cavity of FIV IN complexed with the transferred strand of proviral DNA is sterically consistent with docking of INSTIs.

Both compounds interacted with the two metals within the catalytic cavity. In both cases, the metal-interacting groups were consistent with the pharmacophoric groups described in the 'classic' studies on HIV-1 IN (*i.e*., a γ-keto α-enol carboxylate for the diketo acid, and a β-enol carboxamide plus a lonely pair donor nitrogen for the naphthyridine carboxamide [1,26]). Table 1 summarizes the most important interactions between ligands and FIV IN-DNA complex, considering the residues included in a distance of 5.0 Å starting from the center of the ligand. Of note, interacting residues include FIV IN T59, E85, F114 and N147, which correspond to HIV-1 IN T66, E92, F121 and N155, *i.e*. the aforementioned residues involved in susceptibility to INSTIs.

**Table 1:** Close interatomic contacts between ligands (8,9) and the target.

FIV IN^a^	HIV IN^a^	CHI1019 (**8**)^b^	L-870,810 (**9**)^b^
**D57**	**D64**	X	X
C58	C65	X	X
T59	*T66*	X	X
H60	H67	X	X
E85	*E92*	X	X
T86	T93	X	
**D109**	**D116**	X	X
N110	N117	X	X
G111	G118	X	X
P112	S119	X	
N113	N120	X	X
F114	*F121*	X	X
**E145**	**E152**	X	X
N147	*N155*	X	X
K152	K159		X
C19	C19	X	X
A20	A20	X	X

*a *FIV integrase (IN) residues in close contact with the ligands (5.0 Å cutoff) and equivalent residues in HIV-1 IN. Ligands are numbered as in Fig. 4. The active site residues are shown in bold; HIV-1 residues associated with resistance to IN strand transfer inhibitors are in italics; C19 is a DNA nucleotide base, while A20 is the terminal nucleotide of the 3'- end of 3'-processed viral DNA. Numbering of nucleotides corresponds to that adopted in the crystal structure of transposable DNA bound to Tn5 transposase that was used in the present study to model the FIV proviral DNA. *b *Residues that show close contacts or hydrogen bond interactions with the corresponding ligand are highlighted by a cross. The pose with the highest GOLD score for each compound was considered as the best docking solution.

The best docking solution for L-870,810 obtained in the present study is different from that obtained by one of us in a previous study using a two-metal structure of HIV-1 IN complexed with 5CITEP as a surrogate platform for INSTI docking [22]. That study showed preferential interactions of the β-hydroxy carbonyl group of naphthyridine carboxamides with the metal between D66 and E152. Interactions consistent with coordination of the metal between D66 and D116 were present as well, but were provided by oxygens in the substituents [22]. Similar docking solutions were obtained also in the present study but had lower GOLD fitness scores (data not shown). Differences between the present study and the previous one can be attributable to differences between the predicted folding of FIV IN and the 3D structure of HIV-1 IN, or between the 5CITEP molecule mimicking proviral DNA and the proviral DNA model proposed in the present study. On the other hand, it is possible that both docking poses coexist *in vivo*, given the alternative binding modes crystallographically documented for other ligands.

### *In vitro *activity of integrase inhibitors in FIV-infected cell cultures

**Figure 5:**
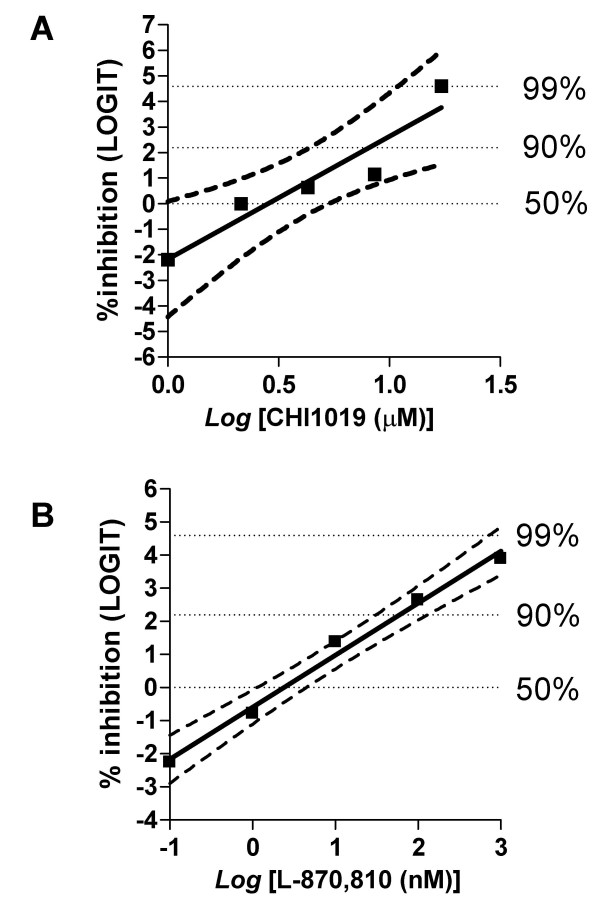
***In-vitro *inhibition of FIV replication by CHI1019 (Panel A) and L-870,810 (panel B)**. MBM cells were infected with FIV-Petaluma (FIV-Pet) in the presence of CHI1019 (panel A) or L-870,810 (panel B), and maintained for seven days in the presence of the inhibitors. FIV replication was quantified by measuring p25 core antigen release in cell culture supernatants. Drug efficacy was assessed as percent decrease in p25 concentrations. Data points represent an average from three independent experiments following appropriate transformations to restore linearity. The solid line is the line best fitting the data points; the dashed curves represent the 95% confidence limits. The EC_50 _values (reported in the main text) were calculated by transposing onto a linear scale the intersection of the regression line (and 95% confidence limits) with the dotted line corresponding to 50% inhibition of viral replication.

If our model for the FIV IN/INSTI interaction is correct, INSTIs designed for HIV-1 should also inhibit FIV replication in cell cultures. For this purpose, feline lymphoblastoid MBM cells were acutely infected with FIV-Pet in the presence or absence of different concentrations of CHI1019 or L-870,810. The NRTI abacavir was used as a positive control for FIV inhibition due to its known anti-FIV effects [7]. As expected, abacavir efficiently abated FIV replication (*P *= 0.0053; *t*-test for regression) with a 50% effective concentration (EC_50_) below 0.625 μM (data not shown). Likewise, CHI1019 inhibited FIV replication in a concentration-dependent manner (*P *= 0.0142; *t*-test for regression) with a calculated EC_50 _of 3.16 μM (1.0–5.6 μM; 95% confidence limits/CL) at seven days post-infection (Fig. 5A). Similar EC_50 _values had previously been reported in HIV-1-infected cell cultures (2.4 μM [31]). The concentration of CHI1019 decreasing MBM cell viability by 50% (CC_50 _≅ 42.8 μM; data not shown) was approximately one order of magnitude higher than the EC_50_, in line with that reported for human lymphoblastoid MT-4 cell line (49.2 μM [31]). The selectivity index of CHI1019 for FIV-Pet was thus calculated to be 13.4. Similar results were obtained using the non-fluorinated analogue CHI1010 (data not shown). Naphthyridine carboxamide L-870,810 also inhibited FIV replication in a concentration-dependent manner (*P *= 0.0005; *t*-test for regression). L-870,810 acted as a more potent inhibitor of FIV replication as compared to the diketo acids, the EC_50 _residing in the low nanomolar range (mean: 2.4 nM; 95%CL: 1.0–4.5 nM Fig. 5B). These results are in line with the EC_50 _values reported in HIV-1 infected cell cultures (ranging from 4 to 15 nM [26]). No toxic effects were observed using L-870,810 at concentrations up to 10 μM. In full agreement with results obtained with HIV-1 [26], the selectivity index of L-870,810 was in the order of approximately 10^4^, making it one of the most potent anti-FIV agents ever tested *in vitro*.

In line with their postulated mechanism of action, CHI1019 and L-870,810 at concentrations up to 10 μM and 1 μM, respectively, did not inhibit FIV p24 production in FL-4 cells harboring copies of integrated FIV DNA (data not shown). We conclude that the test compounds inhibit FIV replication pre-integrationally as effectively as reported for HIV-1. Small differences in the EC_50 _in HIV-1 and FIV assays are likely to be attributed to the different tests and cell lines adopted.

### Quantification by real-time PCR of viral DNA products in the presence of integrase inhibitors

**Figure 6:**
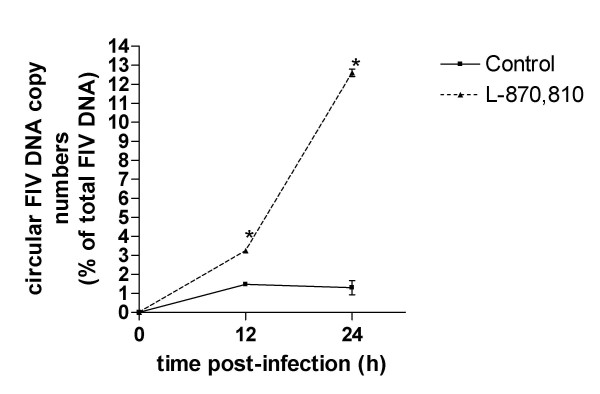
**FIV DNA circle formation in the presence and absence L-870,810**. The relative intracellular content of proviral DNA circular forms is presented as a percentage of the total viral DNA. Means (± SD) from two tests are reported. Asterisks indicate the significant difference (*P *< 0.01) between treatments (no treatment and 1 μM of L-870,810) at the different time points (12 and 24 h post-infection).

If INSTIs indeed inhibited IN strand transfer within the acutely FIV-infected cells, circular forms of proviral DNA should accumulate intracellularly, as previously reported using HIV-1-infected cells [26]. To investigate this effect in FIV-infected cell cultures, we set up and performed quantitative real-time PCR assays to measure total and circular FIV DNA forms [see Additional file 2]. This PCR assay can detect and quantify the total viral DNA (represented by a 153 bp IN CCD fragment), and the circle structure (represented by a 173 bp fragment at the circle junction). The real-time PCR assays developed were found to be reliable and reproducible [see Additional file 3]. To measure the effects of INSTI treatment on viral DNA products, we infected the MBM cells with FIV-Pet in the presence or absence of 1 μM of L-870,810. Intracellular DNA was extracted at 12 and 24 h after infection. Treatment with L-870,810 did not significantly affect the intracellular content of total FIV proviral DNA (*e.g*. 4.73 ± 0.55 × 10^3 ^copies per million cells in untreated controls *vs*. 4.84 ± 0.71 × 10^3 ^in L-870,810-treated cells at 12 h post-infection, means ± S.D., two experiments), thus showing that this drug does not interfere with reverse transcription or any of the steps of FIV replication preceding it. In contrast, the circular proviral DNA increased proportionally over time in L-870,810-treated cells (Fig. 6). This result provides additional evidence that L-870,810 inhibits FIV infection at the level of retroviral integration.

## Conclusion

To sum up, the results of the present study strongly suggest that FIV IN is susceptible to INSTIs designed for HIV-1. There was a good agreement between the results of the bioinformatic analyses of FIV IN and those of the biological assays. These findings may enhance our knowledge of this class of enzymes, which represents a new important target in treatment of HIV-1/AIDS.

Susceptibility of FIV to INSTIs has important implications for continuing research with FIV as an animal model for lentiviral infections. Of course, trials in FIV-infected animals are required before extending the conclusions of the present study to *in-vivo *settings. If *in-vivo *experiments should confirm FIV susceptibility to INSTIs, this animal model could allow studying the long-term effects of drug treatment on viral persistence or emergence of resistant isolates. The FIV model would have the advantage of being low cost and easily accessible.

FIV is not only an interesting animal model for retrovirologists, but is also an important pathogen in veterinary practice. Therefore, the present study may also provide the bases for providing a potential treatment to alleviate disease and prolong survival time of infected pet cats. For example, L-870,810, an INSTI successfully tested in humans, used in combination with NRTIs active on FIV could lead to an ART equivalent for feline AIDS.

## Methods

### Sequences and viral isolates

All amino acid sequences of lentiviral INs were retrieved from the U.S. National Center for Biotechnology Information (NCBI) website [32] except for the *pol *sequences of FIV-M2 and FIV-M3 isolates. FIV-M2 and FIV-M3 were isolated from two naturally infected cats living in Pisa, Italy. Based on *gag *and *env *sequencing, the two viruses were classified as FIV-Fca Clade B [33]. FIV-Fca is the feline lentivirus circulating in domestic cats [28]. By limiting the *in vitro *cultivation in feline lymphoblastoid MBM cells to at minimum (see below), these isolates retained most of the features (*i.e*. high resistance to antibody-mediated neutralization, pathogenicity) typical of the field isolates [34]. For the present study, the genomic DNA of FIV-M2- and FIV-M3-infected MBM cells was extracted with the QIAamp blood kit (Qiagen, Milan, Italy) and PCR-amplified with primers encompassing the whole *pol *gene. Amplicons were then sequenced by cycle sequencing using an automated DNA sequencer (GE Healthcare, Milan, Italy). Primers used for amplification and sequencing and PCR amplification profiles are available upon request by e-mail. Sequences are being submitted to GenBank.

### Phylogenetic analysis

Sequences were aligned using Clustal-X [35], and then the amino acid alignment was manually edited in order to maximize positional homology using the Bioedit program (version 7.0.9.0) [36]. Gaps were removed from the final alignment. Phylogenetic trees were generated with the F84 model of substitution using neighbor-joining method. The statistical robustness and reliability of the branching order within each phylogenetic tree were confirmed with a bootstrap analysis using 1000 replicates. All calculations were performed with PAUP* software, version 4.0b10 (D. L. Swofford, Sinauer Associates, Sunderland, MA) [37].

### Molecular modeling

Reference 3D structures of HIV-1 IN CCD [PDB:1BL3] and Tn5 transposase [PDB: 1MM8] were retrieved from the Protein Data Bank (PDB) [38] through the NCBI website [32]. For homology modeling, target and template sequences were aligned using CLUSTALX. The alignment was then submitted electronically to the Swiss Model server [39], which automatically generates a homology model based on the template structure. Energy computations were done *in vacuo *using the GROMOS96 implementation of the Swiss PDB Viewer (SPDBV) program (Swiss Institute of Bioinformatics) [39]. Energy minimization was carried out by 20 cycles of steepest descent, and minimization stopping when the Δ energy was below 0.05 kJ/mol, as previously described [22]. Hydrogens were added using VEGA ZZ (University of Milan, Italy; freely available at: [40]). The model was then submitted to the MolProbity server [41] for Ramachandran analysis.

To obtain structural alignments, the α-carbons of the highly conserved catalytic triads were initially superimposed using SPDBV, which minimizes the root-mean-square distance (RMSD) between the corresponding atoms using a least square algorithm [39]. Using the default matrix embedded in the program (with open and extended gap penalties of 6 and 4, respectively), the calculation was extended to neighboring atoms until the maximum number of aligned atoms with the lowest RMSD was obtained. The SPDBV software was used to visualize the superimposed structures and transfer selected items from one structure to another. Nucleic acid structures were corrected manually using VEGA. The same program was also used to add hydrogens to the nucleic acids.

The docking platform was further improved using the option' *prepare file for docking programs*' available at the WHAT-IF web interface [42], which performs a small regularization of submitted structures. The protein file was eventually converted to *mol2 *format using Mercury (v. 1.4.2; Cambridge Crystallographic Data Centre/CCDC, Cambridge, UK).

Ligand 3D structures were initially generated as *pdb *files using the CORINA web interface [43], on the basis of the SMILES strings published in the NCBI website. The program VEGA was adopted to assign the correct bond types. The compounds were considered in their keto-enol tautomeric form, since it has been clearly established that these molecules mainly exist in this form in solution (reviewed in: [1]). Moreover, both ionic forms were generated for the carboxylic acid and enol groups of compounds. Using the default parameters in the VEGA program, force fields and charges were assigned according to AMBER and Gasteiger algorithms, respectively, and the molecules were energy-minimized by 50 cycles of conjugate gradients, as previously described [22]. Minimization was stopped when the RMSD between two subsequent solutions was lower than 0.1 Å. Energy minimized ligands were then saved as *mol *files [22].

Automated docking studies were then performed using the genetic algorithm GOLD (Genetic Optimization for Ligand Docking) (v. 3.1; CCDC), according to a protocol previously validated by some of us [20,22]. The binding site was initially defined as all residues of the target within 10 Å from the metal atom coordinated by aspartate residues corresponding to HIV-1 IN D64 and D116, and later automated cavity detection was used. GOLD score was chosen as fitness function and the standard default settings were used in all calculations. For each of the 10 independent genetic algorithm runs, a default maximum of 10,000 genetic operations was performed, using the default operator weights and a population size of 100 chromosomes. Default cutoff values of 2.5 Å for hydrogen bonds and 4 Å for Van der Waals interactions were employed. The two metal ions were set to allow hexavalent coordination according to a Mg^2+ ^type (*i.e*. the metal thought to act as a co-factor *in vivo*). Carboxylate and carboxamide substituents on aromatic rings were allowed to rotate. Early termination was allowed for results differing by less than 1.5 Å in ligand all atom RMSD.

The target/ligand complexes obtained were optimized using the force field CHARMM [44] by two sets of minimizations: the first one was carried out using the steepest descent algorithm with 1000 maximum interactions until the RMSD was 0.1, while the second minimization was performed using the conjugated gradients algorithm, again with 1000 maximum interactions until the RMSD was 0.1.

Post-docking analysis was carried out using SILVER (CCDC).

### Drugs

The synthesis of CHI1010 and CHI1019 was performed as previously reported [31] and summarized in Fig. 4. 5-Chloro-1*H*-indole **(1) **was 3-acetylated **(2) **by reaction with acetyl chloride using diethylaluminum chloride as catalyst and then N-alkylated by treatment with the suitable benzyl bromide in the presence of sodium hydride to give the corresponding 3-acetyl-1-benzyl-1H-indole **(3–4)**. These derivatives were successively condensed with diethyl oxalate and a catalytic amount of sodium methoxide to give ethyl esters **(5–6)**. This reaction was performed under microwave irradiation: reaction times were strikingly reduced (*i.e*. 4 min.), yields were almost quantitative. Finally, deketoesters were converted by basic hydrolysis into the corresponding acids **(7–8)**. L-870,810 (purified powder) was a gentle gift of Merck and Co. (West Point, PA).

### Test for detection of activity of integrase inhibitors *in vitro*

Inhibition of FIV replication was assessed in the feline lymphoblastoid MBM cells, a CD3^+^, CD4^-^, and CD8^- ^T lymphocyte cell line originally established from an FIV-negative and feline leukemia virus-negative cat [45]. Cells were grown in RPMI 1640 medium supplemented with 10% fetal bovine serum, 5 μg of concanavalin A, and 20 U/ml of human recombinant interleukin-2 (Roche Diagnostics, Milan, Italy). Viral stocks of FIV-Pet were obtained from the chronically infected feline T-lymphocyte FL-4 cells [46], as previously described [47].

In the uninfected controls, drug cytotoxicity and CC_50 _values were determined by trypan blue exclusion, by the MTT method and by propidium iodide staining, according to standard techniques previously validated in our hands [48].

Virus inhibition assays were performed in 96-well microplates with 10^5 ^MBM cells and 200 FIV-Pet infectious doses/well. Briefly, MBM cells resuspended in 100 μl of culture medium were mixed with an equal volume of medium containing the virus and decreasing concentrations of CHI1010, CHI1019, L-870,810 or abacavir at which no toxic effects had been observed. Cells were then incubated at 37°C for 4 h. Cells were then washed to remove the excess virus and grown in fresh medium with the above-mentioned drug concentrations. At day 4, 100 μl of supernatant was collected from each well and replaced with fresh medium plus test compounds. Cultures were stopped on Day 7, and virus released in supernatant was monitored for FIV p25 capsid protein content as described using commercially-available FIV p25 ELISA kits (Cell Biolabs, Inc., San Diego, CA), following the manufacturer's instructions. Each drug concentration was tested in triplicate. Inhibition of viral replication was calculated as percent reduction of mean p25 concentration in wells inoculated with FIV and the drug, compared to mean p25 readouts in wells inoculated with FIV alone.

To test the dose-dependence of inhibition of virus or cell growth, serial concentrations of the antiretrovirals were plotted against the percentage-of-inhibition values as previously described [48]. An appropriate transformation such as *Log *or *logit *was used to restore normality. The *logit *of a number *x *between 0 (0%) and 1 (100%) was defined as: *logit x *= *Log *[*x*/(1-*x*)]. The line that best fitted the points was calculated by the least squares method. *t*-tests were used to analyze slope values (*t*-test for regression). The EC_50 _and CC_50 _values, means and 95% confidence limits, were deduced from the regression line and transposed onto a linear scale. Calculations were conducted using the GrapPad software (V. 4.0; GraphPad Software, Inc., San Diego, CA).

### Quantitative real-time PCR assays

To quantitate total and circular proviral DNA, 12 h- and 24 h-old FIV-infected MBM cell cultures (10^6^/sample) were harvested, washed in phosphate-buffered saline, and treated with 500 units of DNaseI (Roche Diagnostics) at 37°C for 1 h prior to DNA extraction. DNAs were prepared by the standard protocol for DNA extraction from cells with the Nucleospin Blood Quick Pure kit (Macherey-Nagel GmbH, Düren, Germany) according to the manufacturer's instructions.

For PCR assays, two different primer pairs were designed from the FIV-Pet nucleotide sequence (accession number M25381). The primer pair 5'-AGGGAACCCACAGTCACAAG-3' (position 4829–4848)/5'-GCCATCCCTCCTATCCTACC-3' (position 4987–4968) and 5'-CTTGAGGCTCCCACAGATACAAT-3' (position 9367–9389)/5'- GTTCGTAAACAGTCCCTAGTCC -3' (position 66–45) allowed the amplification of 159 bp in the *pol *gene (IN core region) and 173 bp of the proviral DNA circle respectively.

A sybergreen real-time PCR assay was set up to detect and quantify the viral DNA using LightCycler instrument (Roche Diagnostics, Germany). To this aim, a recombinant plasmid carrying the 159 bp *pol *fragment obtained from genomic DNA of chronically FIV-Pet infected FL-4 cells, was generated by cloning the amplicon into pGEM T-easy vector (Promega, Madison, WI). PCR reaction was carried out in glass capillary tubes (Roche Diagnostics) containing 150 ng of genomic DNA, 7. 5 μl of 2X commercial ready-to-use PCR master mix sybergreen (QuantiTect sybr green PCR kit, Qiagen, GmbH, Germany), and 0.5 μM of primers (15 μl final volume). Thermal cycling conditions were as follows: initial denaturation at 95°C for 15 min, followed by 45 amplification cycles at 94°C for 15 s, 56°C for 20 s, and 72°C for 20 s. Fluorescence was measured on F1 channel at each extension phase, and the amplification was followed by a melting program, which started at 94°C for 3 s, 65°C for 10 s and then increased to 92°C at 0.1°C/s, with the fluorescence signal continuously monitored on-line.

Ten-fold serial dilutions (from 10^7 ^to 10^2 ^copies) of the recombinant plasmid previously characterized were used as standards in all experiments. Samples, PCR-negative control (ultrapure water PCR grade) and DNA standards were run in parallel and in triplicate.

For the quantitative interpretation of the LightCycler results the "fit point method" algorithm was used, as previously described [49]. A calibration curve was generated from amplification of standard serial dilutions, and threshold cycle (Ct) values were determined and plotted against plasmid copy numbers. Variation over time of the proportion of circular forms of proviral DNA was assessed by Bonferroni's posttest following two-way ANOVA.

## Competing interests

The author(s) declare that they have no competing of interest.

## Authors' contributions

A. Savarino conceived and coordinated the study, did the molecular modeling studies, statistical analyses and drafted the manuscript; M. Pistello conceived and coordinated the assays to measure the antiviral activity *in vitro *and participated in manuscript drafting; D. D'Ostilio, E. Zabogli, and D. Matteucci performed the biological assays for antiviral activity detection; F. Taglia and M. Ciccozzi carried out the phylogenetic analyses and sequence alignments; S. Ferro and A. Chimirri synthesized the CHI1010 and 1019 compounds; L. De Luca computed the energy minimizations of the 3D models; M.L. Barreca conceived the 3D model of lentiviral integrases in complex with Tn5 transposase-derived DNA; A. Ciervo designed and developed the real-time PCR assays and contributed to manuscript drafting; F. Mancini did the molecular biology assays for viral DNA detection and quantitation; M. Bendinelli assessed the validity of the results and supervised the entire study.

## Supplementary Material

Additional file 1 Ramachandran plot for the homology-based model of FIV integrase catalytic core domain. The output of an analysis conducted using MolProbity (see Ref. [41]) is shown. Click here for file

Additional file 2 Sensitivity and reproducibility of the real-time quantitative assay and melting curve profile of specific amplicons. The text describes the experiments devised for validation of the real-time PCR assays adopted in the present study. Click here for file

Additional file 3 Real-time quantitative assay. Sensitivity and reproducibility of the test (Panel A) and melting curve profile (Panel B). Panel A: Graphical representation of the DNA standard curve (ranging from 10^7 ^to 10^2 ^copies per reaction) based on the recombinant plasmid pGEM-T easy vector carrying the specific 159 bp integrase core fragment. The corresponding intra- and inter-assay calculations were done on the basis of the threshold cycles plotted against the logarithm of the copy numbers. The coefficient of variation and the test efficiency were calculated for each point of the standard curve. Panel B: Melting point analysis [fluorescence versus temperature (-d*F*1/d*T*)] and differentiation between the 159 bp integrase fragment and the 173 bp DNA circle amplicon. The box shows the gel analysis of amplicons. Click here for file
