# Supplementary material for: Human immunodeficiency virus integrase inhibitors efficiently suppress feline immunodeficiency virus replication in vitro and provide a rationale to redesign antiretroviral treatment for feline AIDS
Source: Retrovirology. 2007 Oct 30;4:79. doi: 10.1186/1742-4690-4-79 (PMC2244644; doi:10.1186/1742-4690-4-79)
Supplement: Additional file 1 — Ramachandran plot for the homology-based model of FIV integrase catalytic core domain. The output of an analysis conducted using MolProbity (see Ref. [41]) is shown. [file 1742-4690-4-79-S1.pdf]

MolProbity Ramachandran analysis

FIV_PETALUMA_H_MINIMIZED_clean_pdbv3.pdb, model 1

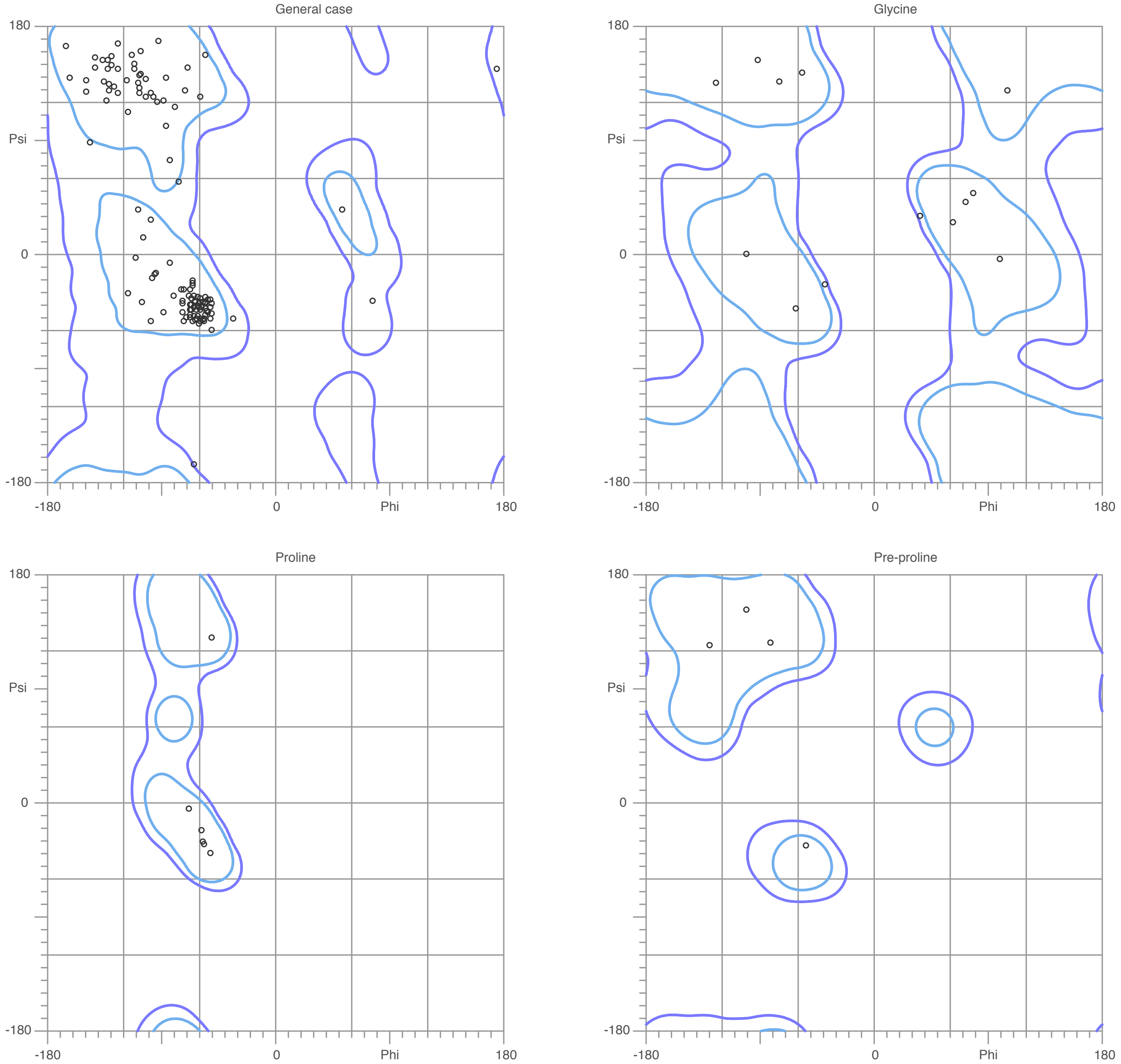

95.3% (141/148) of all residues were in favored (98%) regions.
100.0% (148/148) of all residues were in allowed (>99.8%) regions.

There were no outliers.

http://kinemage.biochem.duke.edu

Lovell, Davis, et al. Proteins 50:437 (2003)
