# Supplementary material for: Human immunodeficiency virus integrase inhibitors efficiently suppress feline immunodeficiency virus replication in vitro and provide a rationale to redesign antiretroviral treatment for feline AIDS
Source: Retrovirology. 2007 Oct 30;4:79. doi: 10.1186/1742-4690-4-79 (PMC2244644; doi:10.1186/1742-4690-4-79)
Supplement: Additional file 2 — Sensitivity and reproducibility of the real-time quantitative assay and melting curve profile of specific amplicons. The text describes the experiments devised for validation of the real-time PCR assays adopted in the present study. [file 1742-4690-4-79-S2.doc]

**Sensitivity and reproducibility of the real-time quantitative assay for FIV total DNA and circular DNA forms and melting curve profile of specific amplicons**

To assess the reproducibility of the assay, the intra- assay and the inter-assay variations were calculated. Each dilution of the DNA standard was measured in triplicate and in 3 independent runs. The percentage of the coefficient of variation (CV %) and the test efficiency were calculated as:

*(SD/mean Ct) x 100*

where SD is the standard deviation, and [10 ^(-1/slope)^] – 1 respectively.

Amplification of serial dilutions revealed a linear detection range from 10^7^ to 10^2^ copies per reaction, with a Pearson’s correlation coefficient (*r*) of approximately 1. The intra-assay variation revealed a CV % ranging from 0.12 to 1.12 with a test efficiency of 95%, while the CV % reproducibility in the inter-assay variation tests ranged from 0.39 to 3.92 with a test efficiency of 90% [see Additional file 3]. Taken together, these findings show that our real-time PCR assay was highly reliable and robust in terms of sensitivity and reproducibility.

The figure shows melting curve analysis profiles that allowed the differentiation between the PCR products of 159 bp DNA fragment core integrase (melting peak at 73.5°C), that correspond to the total proviral DNA, and the 173 bp amplicon (melting peak at 75.5°C), that characterize the proviral DNA circle [see Additional file 3].
