# Supplementary material for: Human immunodeficiency virus integrase inhibitors efficiently suppress feline immunodeficiency virus replication in vitro and provide a rationale to redesign antiretroviral treatment for feline AIDS
Source: Retrovirology. 2007 Oct 30;4:79. doi: 10.1186/1742-4690-4-79 (PMC2244644; doi:10.1186/1742-4690-4-79)
Supplement: Additional file 3 — Real-time quantitative assay. Sensitivity and reproducibility of the test (Panel A) and melting curve profile (Panel B). Panel A: Graphical representation of the DNA standard curve (ranging from 107 to 102 copies per reaction) based on the recombinant plasmid pGEM-T easy vector carrying the specific 159 bp integrase core fragment. The corresponding intra- and inter-assay calculations were done on the basis of the threshold cycles plotted against the logarithm of the copy numbers. The coefficient of variation and the test efficiency were calculated for each point of the standard curve. Panel B: Melting point analysis [fluorescence versus temperature (-dF1/dT)] and differentiation between the 159 bp integrase fragment and the 173 bp DNA circle amplicon. The box shows the gel analysis of amplicons. [file 1742-4690-4-79-S3.ppt]

## Slide 1
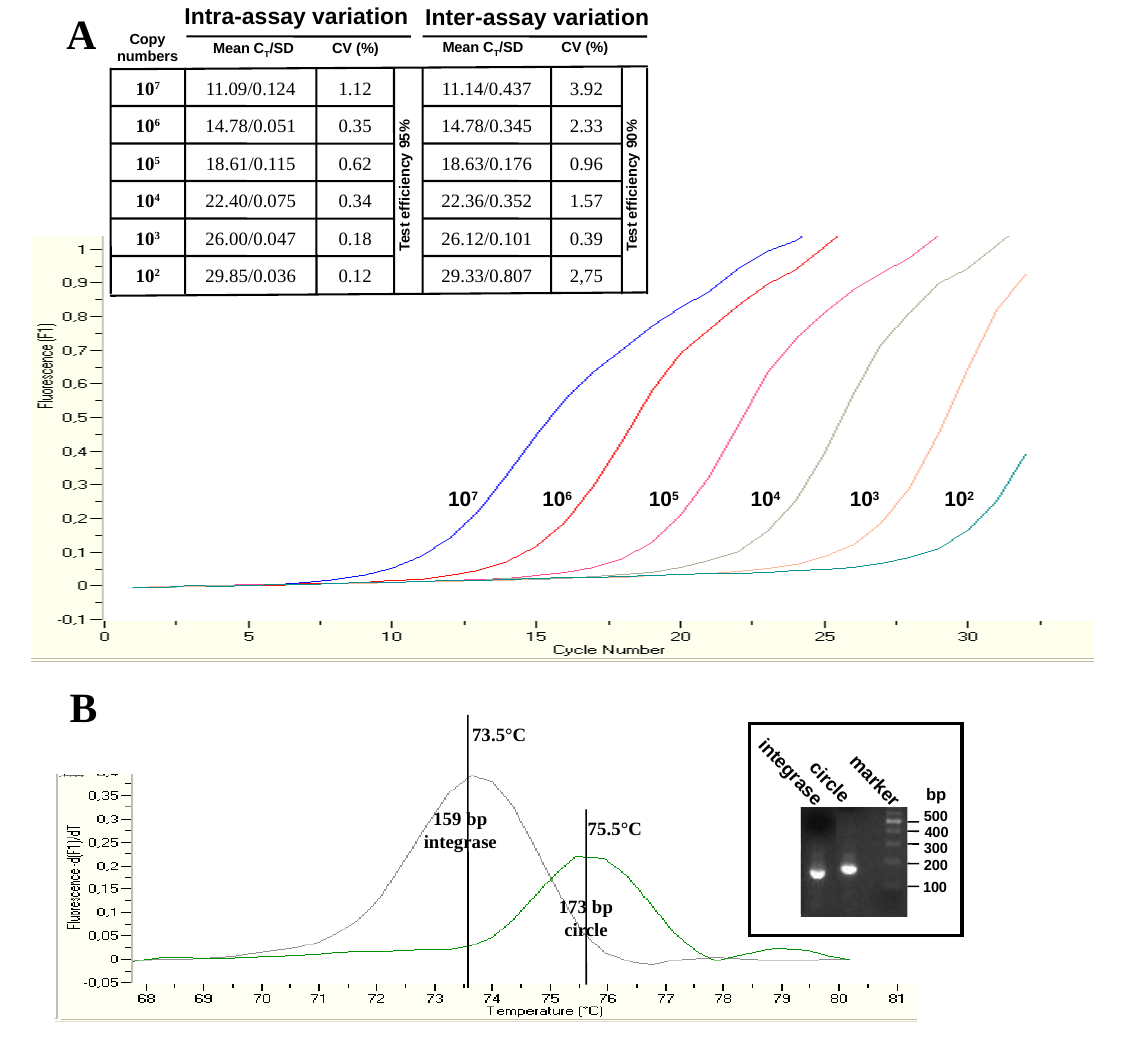

Intra-assay variation
Inter-assay variation
Copy
numbers
CV (%)
Mean CT/SD
CV (%)
Mean CT/SD
107
11.09/0.124
1.12
11.14/0.437
3.92
106
14.78/0.051
0.35
14.78/0.345
2.33
105
18.61/0.115
0.62
18.63/0.176
0.96
Test efficiency 95%
Test efficiency 90%
104
22.40/0.075
0.34
22.36/0.352
1.57
103
26.00/0.047
0.18
26.12/0.101
0.39
102
29.85/0.036
0.12
29.33/0.807
2,75
A
107
106
105
104
103
102
B
73.5°C
integrase
marker
circle
bp
500
400
300
200
100
159 bp
integrase
75.5°C
173 bp
circle
